# Secondary analysis of REPRISE III trial: The Lotus valve’s persistence after withdrawal

**DOI:** 10.21542/gcsp.2023.30

**Published:** 2023-09-30

**Authors:** Susy Kotit

**Affiliations:** Aswan Heart Centre, Aswan, Egypt

## Abstract

Introduction: Aortic stenosis (AS) is the leading heart valve disease in developed countries, often caused by calcific degeneration. In low-and-middle-income countries, it’s primarily due to RHD. Prevalence of AS increases with age and up to 22.8% of those affected over the age of 75. While surgical aortic valve replacement is standard treatment for AS, many older individuals are not ideal candidates. Transcatheter aortic valve replacement (TAVR) offers an alternative. The REPRISE III trial showed the Lotus valve outperformed the CoreValve/EvolutR TAVR valves in various metrics over 2 years. Despite its success and over 10,000 implantations, the Lotus valve was pulled from the market, highlighting the need to understand its long-term outcomes.

Study and results: In the REPRISE III trial, the long-term outcomes of TAVR using the Lotus valve were compared to the CoreValve/EvolutR over 5 years across 55 global centers. Of the participants, 581 (95.7%) used the Lotus valve and 285 (93.4%) used CoreValve/EvolutR. Event rates for all-cause mortality were similar between the groups, but the Lotus valve group had lower rates of disabling stroke and pacemaker implantation. The Lotus valve showed a higher aortic gradient but lower effective orifice area. Additionally, the Lotus valve had reduced mild PVL, valve malpositioning, and the need for a second valve. Both groups showed comparable long-term improvements in heart and cardiomyopathy assessments.

Lessons learned: The REPRISE III analysis highlights the favourable long-term outcomes of the Lotus valve and CoreValve/EvolutR for high-risk surgical patients. These findings underscore the importance of ongoing management post-valve procedure and the potential advantages of the Lotus valve design. Further studies comparing these valves to surgery will inform aortic stenosis management and potentially expand TAVR indications. The future goal is to develop a tissue-engineered living heart valve to improve survival and quality of life.

## Introduction

Valvular heart disease is a leading cause of cardiovascular morbidity and mortality worldwide and the resulting disease burden is only projected to increase in the coming decades^1^. Aortic stenosis (AS) is the most common valvular heart disease in developed countries^2,3^, mainly as a result of calcific degeneration. In low- and middle-income countries it is mainly related to RHD^1^. The prevalence of AS increases significantly with advancing age, affecting up to 22.8% of the population over the age of 75^4–11^ and its impact on public health and healthcare resources is expected to increase due to the general aging of Western populations^3,12,13^ andimproved life expectancy^14^.

Symptomatic severe aortic stenosis is associated with high mortality rates of up to 50% at 1 year^15,16^ with conservative treatment^17^. Surgical aortic-valve replacement remains the standard treatment for aortic stenosis^18^, however, many older patients are not suitable candidates for surgical replacement due to an increased procedure risk^17,19,20^.

Transcatheter aortic valve replacement (TAVR) has emerged as an option for these patients^17,21–28^. Widespread uptake of TAVR has subsequently been seen, with excellent outcomes and increasing procedural scope, even in patients with low surgical risk^21,24,29–33^ showing that this technology is non-inferior or superior to surgical aortic valve replacement^34,35^, inspiring the need for longer-term performance data for TAVR valves.

**Figure 1.**
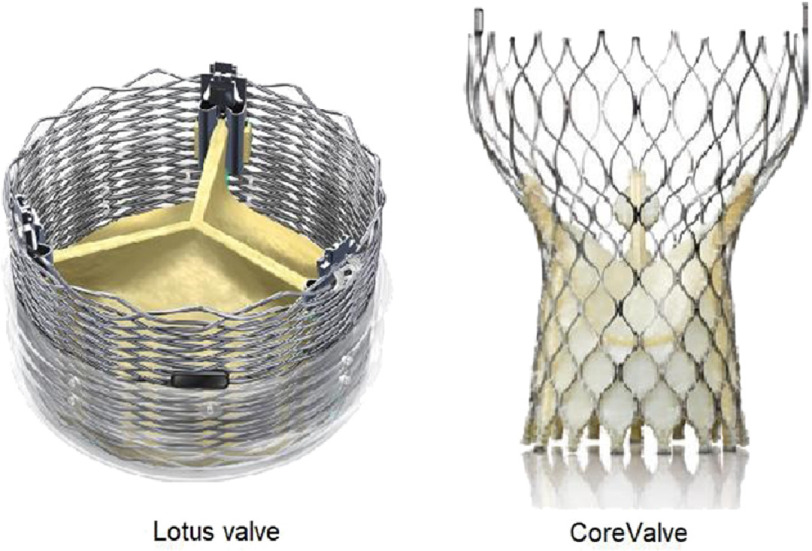
Lotus (Boston Scientific) and the self-expanding CoreValve/ EvolutR (Medtronic) TAVR valves.

The REPRISE III (Repositionable Percutaneous Replacement of Stenotic Aortic Valve Through Implantation of Lotus Valve System—Randomized Clinical Evaluation) trial compared the mechanically expanded Lotus valve (Boston Scientific) and the self-expanding CoreValve/ EvolutR (Medtronic) TAVR valves (Figure 1)^36–38^.

In the studied high-risk population, the mechanically expanded Lotus valve showed no difference in the 30-day primary safety composite endpoint of all-cause mortality, stroke, life-threatening bleeding, stage 2/3 acute kidney injury, and major vascular complications and was superior for the 1-year primary effectiveness end point of all-cause mortality, disabling stroke, and moderate or greater paravalvular leaks (PVLs)^36^. After 2 years, all-cause mortality rates, and mortality or disabling stroke were similar between the Lotus and the CoreValve groups, with disabling stroke, functional class, valve migration, and PVL favouring the Lotus valve whereas valve hemodynamics, thrombosis, and new pacemaker implantation favoured the CoreValve^38^.

Despite the favourable results, the Lotus valve was withdrawn from the market due to the complexity of the manufacturing process and limited commercial uptake, but not before over 10,000 implantations, making its longer-term outcomes of paramount importance.

## The REPRISE III study: Secondary analysis

The pre-specified secondary analysis of the REPRISE III randomized clinical trial compared the long-term outcomes of transcatheter aortic valve replacement (TAVR) with the mechanically expanded Lotus valve (Boston Scientific) and the self-expanding CoreValve/EvolutR (Medtronic) at 5 years in 55 centers worldwide^39^.

Patients with severe native aortic stenosis with a valve area of 1.0 cm^2^ or less (or aortic valve area index ≤ 0.6 cm^2^/m^2^) and a mean pressure gradient of 40 mmHg or greater, or jet velocity of 4.0 m/s or higher, were eligible for enrolment in the initial trial if they had a Society of Thoracic Surgeons predicted risk of mortality of 8% or greater or another indicator of high or extreme risk^36^.

Safety endpoints that were adjudicated by an independent clinical events committee and included mortality, stroke, major vascular complications, new permanent pacemaker implantation, life-threatening or disabling bleeding, myocardial infarction, repeat procedure for valve-related dysfunction, hospitalization for valve-related symptoms or worsening congestive heart failure (New York Heart Association (NYHA) functional class III- indicating a marked limitation in activity due to symptoms, even during less-than-ordinary activity; comfortable only at rest - or class IV, indicating severe limitations; experiences symptoms even while at rest and is mostly bedbound), new onset of atrial fibrillation or flutter, and prosthetic aortic valve thrombosis.

Clinical and echocardiographic assessments occurred annually throughout the 5 years of the trial. Echocardiographic parameters included aortic regurgitation, mean aortic gradient, and effective orifice area (EOA).

Functional status was evaluated using NYHA classification. Health status was evaluated throughout the 5 years using the Kansas City Cardiomyopathy Questionnaire and 12-Item Short Form (SF-12) quality-of-life questionnaire.

The protocol required patients to undergo neurological examinations by a neurology professional at baseline, discharge, 1 year, and after any suspected stroke.

## Results

A total of 912 patients were randomized in the REPRISE III trial, to receive either the Lotus valve ( *n* = 607) or the CoreValve/EvolutR ( *n* = 305). The mean (SD) age of the cohort was of 82.8 (7.3) years and included 463 women (50.8%) and 449 men (49.2%)^36^.

The mean (SD) Society of Thoracic Surgeons risk scores was similar between the Lotus valve and CoreValve/EvolutR cohorts (6.7% and 6.9%). Symptomatic aortic stenosis (NYHA functional class III or IV) was present in 71.3% of the Lotus valve group and 67.9% of the CoreValve/EvolutR group.

At 5 years, the secondary analysis included 581 patients (95.7%) who received the Lotus valve and 285 patients (93.4%) who received the CoreValve/EvolutR and either had an adverse event, or completed the 5-year clinical follow-up. The remaining 46 patients did not have an adverse event and did not complete the 5-year follow-up needed for inclusion in the analysis.

**Figure 2.**
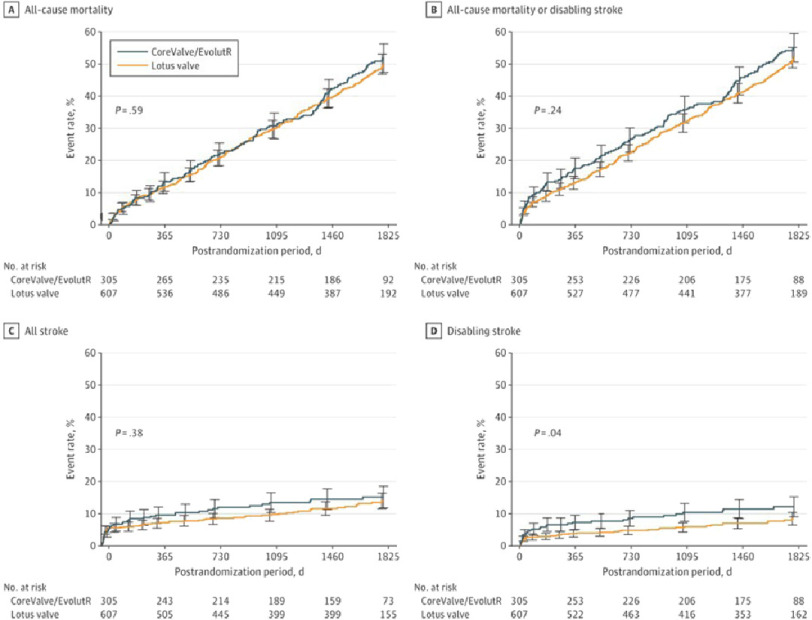
Cumulative event curve for death and stroke.

Clinical Outcomes at 5 Years showed cumulative event rates for all-cause mortality and all-cause mortality or disabling stroke of 50.9% and 52.8% in the Lotus valve group and 52.8% and 56.0% in the CoreValve/EvolutR group ( *P* = 0.59 and *P* = 0.24), respectively. The overall cumulative event rates for stroke and disabling stroke were 14.1% and 8.3% in the Lotus valve group and 15.3% and 12.2% in the CoreValve/EvolutR group ( *P* = 0.38 and *P* = 0.04) (Figure 2).

Cumulative event rates for permanent pacemaker implantation were 38.9% in the Lotus valve group and 27.3% in the CoreValve/EvolutR group ( *P* < 0.001) with most new pacemakers received within the first year of follow-up (3.5% of patients in the Lotus group compared with 6.7% of patients in the CoreValve/EvolutR group) ( *P* = 0.04).

Cumulative event rates for repeat procedures were 1.8% in the Lotus valve group and 2.9% in the CoreValve/EvolutR group ( *P* = 0.09).

The proportion of patients with prosthetic aortic valve thrombosis was 5.8% in the Lotus valve group and 1.8% in the CoreValve/EvolutR group ( *P* = 0.007) which was generally detected during routine echocardiographic follow-up as an increase in the transvalvular gradient. The occurrence of endpoints is summarized in Table 1.

**Table 1:** Endpoints at 5 years.

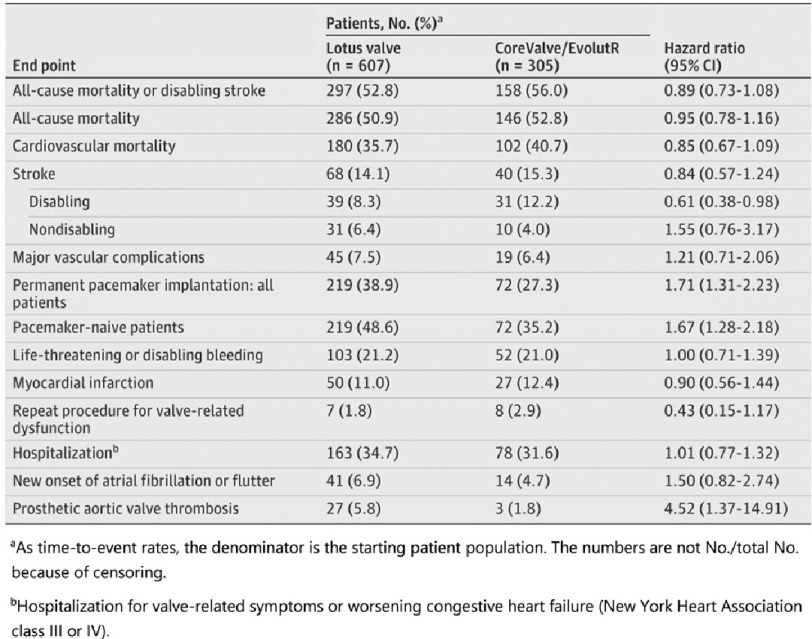

At 5 years, incidences of major vascular complications, life-threatening or disabling bleeding, myocardial infarction, rehospitalization, and new onset of atrial fibrillation or flutter were not significantly different between the valve cohorts.

**Figure 3.**
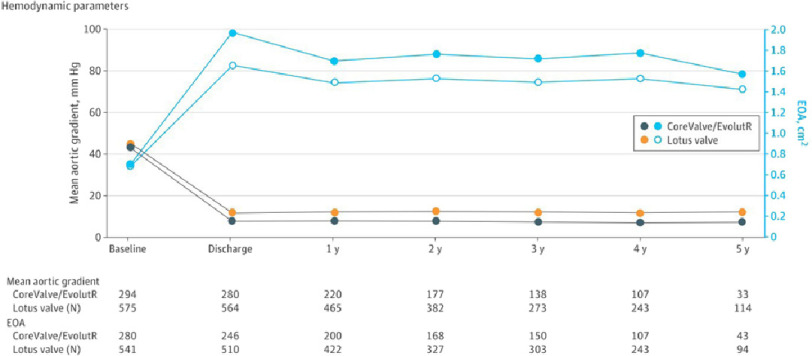
Mean aortic gradient (mmHg) and effective orifice area (EOA) from baseline to 5 years in CoreValve/EvolutR and Lotus valve.

During the study, the mean EOA increased from 0.69 cm^2^ at baseline to 1.65 cm^2^ at discharge remaining at 1.42 cm^2^ at 5 years in the Lotus valve compared to 0.70 cm^2^ at baseline to 1.96 cm^2^ at discharge and 1.57 cm^2^ at 5 years in the CoreValve/EvolutR cohort. The mean aortic gradient decreased from 44.6 mmHg at baseline to 12.2 mmHg at discharge and 12.6 mmHg at 5 years in the Lotus valve compared to 43.9 mmHg to 8.2 mmHg at discharge and 7.8 mmHg at 5 years in the CoreValve/EvolutR (Figure 3).

The mean EOA was significantly lower (1.42 *vs* 1.57 cm^2^; *P* < 0.001) and the mean aortic gradient (12.64 *vs* 7.79 mmHg; *P* < 0.001) was significantly higher in the Lotus valve group compared to the CoreValve/EvolutR group at each follow-up time point throughout the 5 years.

**Figure 4.**
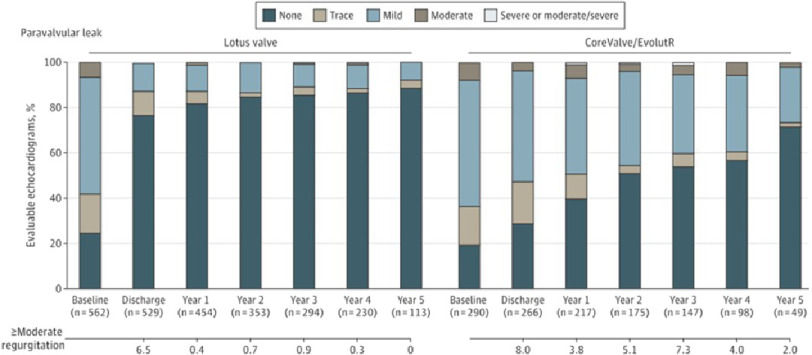
Paravalvular leak across the cohorts from baseline to 5 years.

At 5 years of follow-up, mild PVL was less frequent with the Lotus valve compared with the CoreValve/EvolutR (7.8% *vs* 23.1%; *P* = 0.006), and moderate or greater PVL occurred at a similar rate between cohorts (0% *vs* 1.9%; *P* = 0.31) (Figure 4).

The Lotus valve resulted in less frequent malpositioning (0% *vs* 2.6%; *P* < 0.001) and a decrease in the required use of a second valve (1.0% *vs* 3.8%; *P* < 0.001) during the procedure.

**Figure 5.**
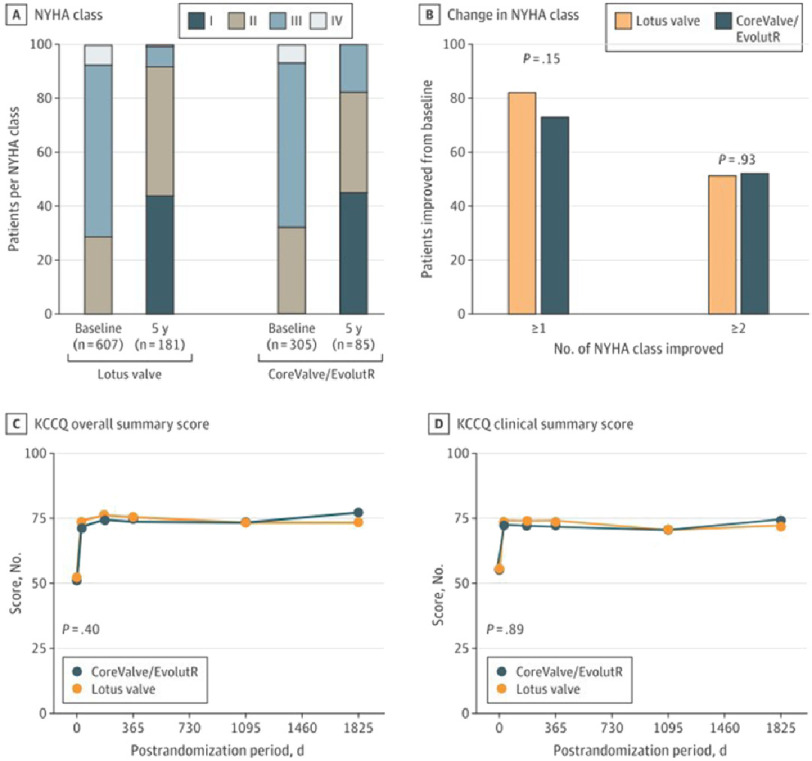
Symptom status from baseline to 5 years.

Of the patients who survived, 91.7% who received the Lotus valve and 82.4% who received the CoreValve/EvolutR had a NYHA functional class of I (indicating no limitation of physical activity) or class II (indicating slight limitation of physical activity) at 5 years. Patients in both the Lotus valve and CoreValve/EvolutR groups improved from baseline by 1 or more NYHA classes (81.8% *vs* 74.1%; *P* = 0.15) or by 2 or more NYHA classes (26.5% *vs* 27.1%; *P* = 0.93). Assessment of the functional status of patients using the Kansas City Cardiomyopathy Questionnaire showed no significant differences between the 2 cohorts at any time point (Figure 5).

## Discussion

Secondary analysis of the REPRISE III trial found that patients treated with the Lotus valve had similar mortality rates, fewer disabling strokes, and similar repeat procedures compared with those who received the CoreValve/EvolutR. Hemodynamics, health, and functional status were maintained long term in both groups, concluding that, at 5 years, the clinical outcomes of the Lotus valve were comparable to those of the CoreValve/EvolutR and that the Lotus valve was safe and effective.

The Lotus valve was designed to address important limitations of other TAVR valves, most notably related to paravalvular regurgitation^40,41^ resulting in a lower rate of malpositioning and the need for a second valve during the procedure. Although at 5 years the results for moderate and severe paravalvular leak were not significantly different between the valves studied, the incidence of mild paravalvular leak was significantly lower in the Lotus group. This is mainly due to the design and mechanism of deployment of the Lotus valve which allowed for full repositioning and resheathing, even in the completely expanded position, and provided hemodynamic stability for the patient enabling the operator to determine the optimal position and the need for repositioning.

In addition, the presence of an adaptive polyurethane sealing membrane around the lower part of the outer surface of the nitinol frame - designed to fill any potential space between the native annulus and prosthesis - minimizes paravalvular leakage.

The Lotus valve cohort had higher aortic gradients, more new pacemaker implantations, and a higher incidence of prosthetic aortic valve thrombosis events, although without increased incidence of stroke associated with prosthetic aortic valve thrombosis. The number of patients with hemodynamic data at 5 years was low, which may have influenced the results on the effective orifice area which was lower than expected in the CoreValve/EvolutR cohort. Importantly, over 5 years, mean transvalvular gradients remained stable among patients in both groups and within the range observed in previous studies^42–45^.

Myocardial infarction exceeded 11% in both groups, and new onset of atrial fibrillation or flutter was found in 4.7% of the patients. In addition, hospitalization for valve-related symptoms or worsening congestive heart failure (New York Heart Association class III or IV) was common ( ≥31.6%). Over one-half of the patients died and about 10% had a disabling stroke during the 5-years of follow up, regardless of the valve used. Although it should be noted this study was performed on a high-risk group, whose mortality rates are up to 50% at 1 year^15,16^ with conservative treatment^17^ and who are not suitable candidates for surgical replacement due to an increased procedure risk^17,19,20^.

Importantly, the results presented in the REPRISE III trial may not be applicable to other patient populations as the study included only patients with the aforementioned high surgical risk. Furthermore, the two valves studied in the trial are no longer on the market or have largely been replaced by newer-generation valves. The valves currently being implanted differ in mechanism and technique, which could impact safety, efficacy, and durability compared to the valves studied.

Overall, the findings of the REPRISE III secondary analysis add important information regarding long-term outcomes among patients with earlier iterations of TAVR valves, which seem favorable for both the Lotus valve and CoreValve/EvolutR, with the maintenance of hemodynamics, health, and functional status in patients contra-indicated for surgery due to high procedural risk.

Nevertheless, the results accentuate the need to take into account the early and very late risks of new conduction disorders, pacemakers, compromised coronary access, or coronary occlusion and show that the consequences of aortic disease are lifelong and require considerations for lifetime management even after corrective valve procedure. In addition, there is a need for further improvement of this revolutionary technique. Design benefits found in the Lotus valve should be studied and applied in future valve design for improvement of prognosis and procedure outcomes.

Long-term results are needed in lower-risk populations, who are typically younger, with less concomitant disease and risk factors, in comparison to surgical results. Analysis of the management of aortic stenosis and formulation of revised guidelines for the standard care of patients across the clinical spectrum is essential, possibly with the extension of indication of TAVR to patients at lower risk, based on rigorous patient selection and integration of aortic stenosis management strategies throughout patient’s lifetime, for optimized outcomes.

The ultimate goal, however, is the development of a tissue-engineered living heart valve to optimize valve replacement, especially in children, enhancing survival, long-term outcomes, and quality of life^46–48^.

### Lessons learned

The long-term outcomes of the Lotus valve and CoreValve/EvolutR seem favourable, with the maintenance of hemodynamics, health, and functional status in patients contra-indicated for surgery due to high procedural risk.

The findings of the REPRISE III secondary analysis add important information regarding long term outcomes among patients with earlier iterations of TAVR valves and accentuate the need for lifetime management after corrective valve procedure as well as further improvement of the technique through implementation of design benefits seen in the Lotus valve.

Long-term results across the severity of disease and procedural risk, in comparison to surgery, are needed for the analysis of the management of aortic stenosis and formulation of revised guidelines for the standard care of patients along the clinical spectrum, possibly with the extension of indication of TAVR to patients at lower risk.

Ultimately, the development of a tissue-engineered living heart valve will optimize valve replacement and enhance survival, long-term outcomes and quality of life.
